# Analysis of the effects of exposure to acute hypoxia on oxidative lesions and tumour progression in a transgenic mouse breast cancer model

**DOI:** 10.1186/1471-2407-8-151

**Published:** 2008-05-28

**Authors:** Tuula M Kalliomäki, Gordon McCallum, Sarah Jane Lunt, Peter G Wells, Richard P Hill

**Affiliations:** 1 Applied Molecular Oncology Division, Ontario Cancer Institute/Princess Margaret Hospital, Canada; 2 Department of Medical Biophysics, University of Toronto, 610 University Avenue, Toronto, Ontario, M5G 2M9, Canada; 3 Radiation Oncology, University of Toronto, 610 University Avenue, Toronto, Ontario, M5G 2M9, Canada; 4 Department of Pharmaceutical Sciences, University of Toronto, 144 College Street, Toronto, Ontario, M5S 3M2, Canada

## Abstract

**Background:**

Tumour hypoxia is known to be a poor prognostic indicator, predictive of increased risk of metastatic disease and reduced survival. Genomic instability has been proposed as one of the potential mechanisms for hypoxic tumour progression. Both of these features are commonly found in many cancer types, but their relationship and association with tumour progression has not been examined in the same model.

**Methods:**

To address this issue, we determined the effects of 6 week *in vivo *acute hypoxic exposure on the levels of mutagenic lipid peroxidation product, malondialdehyde, and 8-oxo-7,8-dihydro-2'-deoxyguanosine DNA (8-oxo-dG) lesions in the transgenic polyomavirus middle T (PyMT) breast cancer mouse model.

**Results:**

We observed significantly increased plasma lipid peroxidation and 8-oxo-dG lesion levels in the hypoxia-exposed mice. Consumption of malondialdehyde also induced a significant increase in the PyMT tumour DNA lesion levels, however, these increases did not translate into enhanced tumour progression. We further showed that the *in vivo *exposure to acute hypoxia induced accumulation of F4/80 positive tumour-associated macrophages (TAMs), demonstrating a relationship between hypoxia and macrophages in an experimental model.

**Conclusion:**

These data suggest that although exposure to acute hypoxia causes an increase in 8-oxo-dG lesions and TAMs in the PyMT tumours, these increases do not translate into significant changes in tumour progression at the primary or metastatic levels in this strong viral oncogene-driven breast cancer model.

## Background

Hypoxia in tumours is a predictor of poor patient survival. This has been shown most extensively in soft tissue sarcoma, and in cervical and head and neck carcinoma patients where tumour hypoxia, as assessed by partial pressure of oxygen (pO_2_) measurements, is predictive of metastatic and/or disease-free survival [1]. In breast cancer patients the role of hypoxia as a predictor for survival has been demonstrated through immunohistochemical detection of hypoxia-dependent markers, such as hypoxia-inducible factor-1α (HIF-1α) and carbonic anhydrase IX (CAIX) [2,3].

The underlying mechanisms for hypoxia-mediated tumour progression are not well understood. Examinations of the effects of different types of tumour hypoxia on metastatic progression have suggested acute (cycling) hypoxia to be more important than chronic (diffusion limited) hypoxia [4,5]. A potential association between acute hypoxia and genomic instability has been suggested by Comet assay data, which showed significantly increased numbers of single-strand DNA breaks in tumour samples from mice exposed to acute hypoxia compared with samples from mice exposed to chronic hypoxia (Cairns and Hill, unpublished data, 2004). Similar data has been reported in tumour cells exposed to increased number of hypoxic cycles *in vitro*, which resulted in increased number of DNA mutations, the frequency and types of which resembled those found in the same cells grown as tumours *in vivo *[6]. *In vitro *hypoxia exposure can also mediate larger scale genomic instability by increasing fragile site induction, gene amplification and cellular DNA content [7-9].

In some cases genomic instabilities in hypoxic tumours have been attributed to hypoxia-mediated inhibition of various DNA repair mediators from the homologous recombination, mismatch repair and non-homologous end joining DNA repair pathways [10-13]. However, the role of reactive oxygen and nitrogen species (ROS, RNS) in tumour progression as mediators of a "mutator phenotype" is also well documented [14,15] and acute hypoxia is known to generate these species through multiple systems including mitochondria, xanthine oxidase, ferrylhemoglobin, NADPH oxidase and thymidine phosphorylase [16,17]. Upon reaction with DNA, free radicals can generate mutagenic DNA lesions, such as 8-oxo-dG, that cause GC→TA transversions and structural changes compromising the transcriptional fidelity of the genome [18]. DNA damage can also be induced indirectly through free radical reaction with polyunsaturated fatty acids of cellular membranes and formation of lipid peroxidation products such as, isoprostanes, 4-hydroxynonenal and malondialdehyde (MDA), shown to induce frame-shift mutations and base pair substitutions [19,20].

Macrophages (MØs), which have been reported to be attracted to hypoxic tumour areas, are also a source of mutagenic superoxide (O_2_^-^), nitric oxide (NO) and peroxynitrite (ONOO^-^) species [21,22]. In tumours, the MØs can promote tumour cell angiogenesis, invasion and survival by up-regulating genes such as GLUT-1, VEGF, CXCR4 and MIF [23] and high levels of these tumour-associated macrophages (TAMs) can predict poor patient survival [24]. In support of the concept of TAM-mediated tumour progression, Pollard and colleagues showed that reducing the number of TAMs in the polyoma middle T (PyMT) breast cancer model by crossing it to op/op null mice with a mutation in the colony stimulating factor-1 (CSF-1) gene, inhibited metastasis formation [25]. Further studies in the same model demonstrated that tumour cell movement and intravasation occur most frequently when the tumour cells are within 20 μm of a perivascular TAM [26].

In this study we examined the effect of acute (cyclic) hypoxia on tumour progression in relation to oxidative stress, TAMs and genomic instability in the MMTV-PyMT model. We demonstrated significantly increased levels of 8-oxo-dG lesions and TAM content in tumours from mice exposed to acute hypoxia but these increases did not translate into significant changes in tumour progression.

## Methods

### Animals

A breeder pair of mice with a PyMT transgene under the MMTV promoter (MMTV-PyMT^634Mul^), originally described by Guy *et al.*, was obtained from Dr. Rama Khokha (Princess Margaret Hospital, Ontario Cancer Institute, Toronto, Canada) [27]. The mice were bred and maintained at the Animal Resource Centre of the Ontario Cancer Institute in compliance with the guidelines of the Canadian Council on Animal Care. Tissue samples were obtained at 2 weeks-of-age and screened for the presence of the PyMT transgene using the following primers (forward) 5' GGA AGC AAG TAC TTC ACA AGG 3' and (reverse) 5' GGA AAG TCA CTA GGA GCA GGG 3'. Mice were weaned at 3 weeks-of-age. All experiments were conducted with heterogeneous, virgin MMTV-PyMT or littermate wild type (WT) mice.

### Hypoxia, malondialdehyde and 2-nitropropane treatments

For acute hypoxia (AH) experiments, mice were treated 5 times a week for 6 weeks, from 3 to 9 weeks-of-age. The hypoxia exposure consisted of 12 cycles of 10 min of air, followed by 10 min of 7% oxygen, balance nitrogen for a daily total of 4 hours, in airtight chambers as described previously [4]. Air control (AC) animals were exposed to a daily total of 4 hours of air in separate sealed chambers and cage control (CC) animals were left in their cages untouched. For HPLC and immunohistochemical analyses, mice were killed within 1 hour following the last gassing exposure at the age of 9 weeks to ensure similar tumour development for mammary tissues from mice treated for 4 hours/day during 1 day, 5 days or 6 weeks. For the tumour progression studies, the mice were left in their cages following the 6-week exposure until they reached the age of 88 days at which time the experiment was terminated and primary tumour growth and metastases were assessed. For determination of tumour hypoxia levels, mice were injected intraperitoneally (10 mM, 0.01 mL/g) with the exogenous hypoxia marker, EF5 ([2-(2-nitro-1*H*-imidazol-1-yl)-*N*-(2,2,3,3,3-pentafluoropropyl acetamide], Dr. Cameron Koch, University of Pennsylvania), 1 hour into the 4 hour gassing exposure during a reoxygenation cycle and killed within 1 hour of the last hypoxic cycle.

Animals treated with the lipid peroxidation product, malondialdehyde, (MDA, Sigma-Aldrich) received acidified water with 10 mM MDA, replaced twice a week while control animals (MDA-CC) received normal acidified water. The drug concentration was based on a measured average daily water consumption of 3.75 mL/mouse/day and calculated to give an approximate daily dose of 250 mg/kg [28]. Mice received the treated water for 6 weeks, from 4 to 10 weeks-of age at which point they were killed if used for HPLC analysis or left to develop their tumours to the age of 81 days if used for tumour progression analysis. An oxidizing agent, 2-nitropropane (2-NP, Sigma-Aldrich), reported to increase the number of 8-oxo-dG lesions in mice, was used as a positive control for the HPLC analyses [29]. It was administered intraperitoneally at 167 mg/kg in sterile olive oil vehicle and the mice were sacrificed 24 hours after a single dose at the age of 81 days.

### Immunohistochemistry (IHC)

Snap frozen tumour tissue from hypoxia-exposed mice was used for IHC staining of markers for macrophages (F4/80; ab6640: 1/300 1 hr R/T; Abcam Inc., Cambridge, MA), base excision repair (APE/Ref-1; ab82: 1/100 O/N 4°C; Abcam Inc., Cambridge, MA), hypoxia (EF5; ELK3-51: 1/50 O/N R/T; Dr. Cameron Koch, University of Pennsylvania) and vasculature (CD31; MEC13.3: 1/500 1 hr R/T; BD Pharmingen) according to manufacturer's instructions. Total area of positive staining in tumour sections, (1 per tumour, of approximately 43 mm^2^) with areas of necrosis and connective tissue excluded, was quantified with the positive pixel algorithm by Aperio ImageScope (Aperio Technologies, Vista, CA). Inter-mouse variability of EF5 staining was quantified in 31 tumours from 11 different mice.

### Assessment of primary and metastatic tumour burdens

In AH experiments primary tumour burden and the number of lung metastases were determined in 88-day-old mice. This time was reduced by a week to 81 days for MDA experiments to reduce the tumour burden prior to sacrifice since the MMTV-PyMT mice develop multiple primary tumours. The primary tumour burden was obtained by multiplying tumour length × tumour width and summing the individual tumour values of each mouse. Lungs were fixed in 10% neutral buffered formalin for 48 hours and then transferred to 70% ethanol. Macroscopic lung metastases in individual lung lobes were scored under a dissecting microscope. Micrometastases in each pair of lungs were summed from 4 sections (separated by 100 μm) stained with haematoxylin and eosin (H+E).

### Lipid peroxidation (TBARS) and antioxidant reductive capacity assays

Heparinized blood from hypoxia- and control-exposed mice was centrifuged at 6000 rpm for 1 min. The separated plasma was divided into 2 aliquots to prevent repeated freezing and thawing of the sample and stored at -80°C until analysis. Plasma samples were analysed with the thiobarbituric acid reactive substance (TBARS) assay, which measures MDA by reaction with thiobarbituric acid to form a red product that was quantified spectrophotometrically. MDA was quantified in 150 μL of plasma with 5 μl of 0.1 M butylated hydroxytoluene (BHT, Sigma-Aldrich). Following the addition of 62.5 μL of pure glacial acetic acid and 62.5 μL of 0.67% thiobarbituric acid (Sigma-Aldrich), the samples were incubated at 50°C for 2 hours and cooled to room temperature for extraction of the coloured reaction product with 1 mL of 1-butanol, which was read with a spectrophotometer at 535 nm [30].

The antioxidant status of the plasma from AH and AC exposed mice was assayed with an antioxidant reductive capacity assay (NWK-ARC01; Northwest Life Science Specialties, Vancouver, WA). This 96-well assay is based on the ability of sample antioxidants to reduce Cu^++ ^to Cu^+^, which reacts with bathocuproine to form a coloured product, read at 492 nm. Plasma samples from 10–11 mice were assayed in triplicate.

### Quantification of 8-oxo-7,8-dihydro-2'-deoxyguanosine lesions in DNA

#### DNA isolation and purification

The DNA extraction protocol was based on work published by Ravanat *et al. *[31]. Briefly, the snap frozen tumour samples were thawed on ice, homogenized with 1 mL of lysis buffer A (10 mM Tris-HCl, 320 mM sucrose, 5 mM MgCl_2_, 0.1 mM desferroxamine, 1% Triton X-100, pH 7.5) and centrifuged at 1,000 g for 10 min to isolate the nuclear pellet. The pellet was washed once in lysis buffer A and resuspended into 200 μL of buffer B (10 mM Tris-HCl, 1% w/v SDS, 5 mM EDTA-NA_2_, 0.15 mM desferroxamine, pH 8.0) using a plastic Kontes Pellet Pestle^®^. RNA was digested by adding 13.3 μl of a 50 U/mL RNase A and 100 U/mL RNase T solutions for 1 hr at 50°C. A proteinase K solution (13.3 μl; 5 mg/mL) in 10 mM Tris/1 mM EDTA, pH 7.4 was added for protein digestion at the same incubation conditions. Prior to the DNA extraction, the samples were centrifuged at 10,000 g for 10 min to remove any undigested tissue. DNA was precipitated with 0.3 mL of Buffer C (40 mM Tris-HCl, 7.6 M NaI, 20 mM EDTA-NA_2_, 0.3 mM desferroxamine, pH 8.0) and 0.5 mL of 100% isopropanol. The precipitate was centrifuged at 10,000 g and washed 4 times with 70% ethanol. The DNA pellet was resuspended in 20 mM Na-acetate buffer with 0.1 mM desferroxamine, pH 5.2. Diluted DNA samples were digested with 5 U of nuclease P1 prepared in 20 mM Na-acetate buffer. The pH was adjusted to 8.5 with 1 M Tris-HCl buffer and the DNA hydrolyzed to nucleosides by incubation with 6 U of calf intestine alkaline phosphatase (Roche Diagnostics, Laval, PQ) for 1 hr at 37°C. The suspension was transferred to Microcon^® ^YM-10 spin columns (Millipore, Mississauga, ON) and centrifuged at 10,000 g for 30 min, filter-sterilized and injected for HPLC analysis.

#### HPLC conditions

The HPLC system (200 series, Perkin Elmer, Woodbridge, ON) consisted of a 0.2 μm mobile phase filter, a vacuum degasser, an isocratic HPLC pump set at 1.0 mL/min, an autosampler with a 200 μL sample loop and a Spherisorb^® ^ODS-2 guard column (10 × 4.6 mm i.d., 5 μm, Waters, Mississauga, ON), and 2 Spherisorb^® ^ODS-2 analytical columns (150 × 4.6 mm i.d., 5 μm, Waters, Mississauga, ON) connected in series. The mobile phase consisted of 7.5% methanol, and 50 mM sodium phosphate buffer, pH 5.5, filtered through a 0.2 μm filter (Millipore, Mississauga, ON). The 8-oxo-dG was detected with an electrochemical detector (Coulochem II 5200A, ESA, Chelmsford, MA) with a PEEK-filter protected analytical cell (5010, ESA; 10nA; screen electrode +100 mV; analytic electrode, +400 mV). The dG was detected with a UV detector (200 series, Perkin Elmer, Woodbridge, ON) set at 280 nm. Data were recorded using a NCI 902 dual detector interface and analyzed with Totalchrom Workstation Ver 6.3.1 software (Perkin Elmer, Woodbridge, ON). Levels of 8-oxo-dG and dG in samples were quantified via calibration curves with authentic standards.

### Statistical analysis

For non-parametric data sets, such as those for metastases, the Kruskal-Wallis test with Dunn's correction for multiple comparisons was performed on experiments with 3 or more groups and the Mann-Whitney test was performed on experiments with 2 groups. For parametric data sets with equal variances, such as those for tumour burden, ANOVA with Bonferroni correction was performed on experiments with 3 or more groups and Student's t-test was performed on experiments with 2 groups. Mixed modelling was used to correct for inter-experimental variability in repeat TBARS assays.

## Results

### Lowering of tumour oxygenation with in vivo acute hypoxia gassing regime

**Figure 1:**
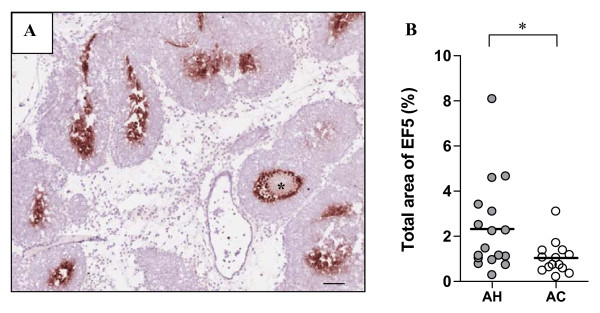
**Levels of EF5 following 4 hours of *in vivo *acute hypoxia exposure**. (**A**) An H+E stained tumour section from a PyMT mouse tumour with EF5 positive hypoxic areas stained in brown, necrotic tumour core is marked with an asterix. Scale bar: 100 μm. (**B**) Quantification of total EF5 positive staining area (%) in 31 tumours from 11 PyMT mice exposed to AC or AH conditions for 4 hours showed significantly greater median levels of hypoxia in mice exposed to AH than in mice exposed to AC (P < 0.05; *).

The hypoxia marker EF5 was used to identify hypoxic areas in the MMTV-PyMT tumours (Figure 1A). Although the overall values of EF5 positive areas were relatively low, the total median EF5 positive area was significantly greater in tumours from mice exposed to 6 weeks of AH than AC gassing (Figure 1B; P < 0.05). Interestingly, inter-tumour variability in EF5 positive areas of individual animals was substantial, varying from 0.3% to 8.1% per mouse.

### Oxidative stress and genomic instability in hypoxia- and MDA-treated mice

**Figure 2:**
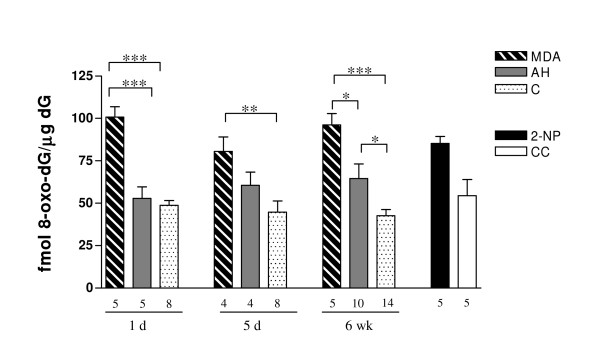
**Tumour 8-oxo-dG lesion levels following oxidative stresses**. Mutagenic 8-oxo-dG lesions quantified at 1 day time point resulted in significant changes between MDA and AH and MDA and C (P < 0.001; ***) groups. Following 6 weeks of treatment the lesion levels were significantly increased in AH mice relative to AC mice (P < 0.05; *). Legends: MDA = malondialdehyde; AH = acute hypoxia; C = pooled controls from air control and PBS injected groups; 2-NP = positive control, 2-nitropropane; CC = cage control. Bars: mean with SEM. Number of tumours used for each group is indicated below the bars.

To determine the extent of oxidative stress induced by the gassing, plasma was obtained from transgenic and WT mice following the 6-week gassing regime. Mean plasma TBARS levels were greater in mice exposed to AH than in animals exposed to AC in 3 separate experiments, a difference that was shown to be significant with mixed modelling analysis (P < 0.0005). TBARS levels in mice drinking MDA-supplemented water were also significantly increased relative to both gassing groups (Table 1). Oxidative stress was also assessed by HPLC quantification of 8-oxo-dG lesions. Tumour samples from mice exposed to AH showed an increasing difference relative to AC mice over the 1 day, 5 day and 6 week exposures. The difference was significant by the 6 week time point (P < 0.05). MDA consumption increased tumour 8-oxo-dG lesions significantly at all time points (Figure 2).

**Table 1:** TBARS plasma lipid peroxidation levels following oxidative stresses

	**Absorbance values (Arbitrary scale)**			
	
	**Air Control (AC)**	**Acute Hypoxia (AH)**	**Ratio (AH/AC)**	**MDA treatment**
**Expt 1 (n = 4–5)**	0.080+/-0.005	0.101+/-0.007	1.27	0.519+/-0.066
**Expt 2 (n = 7)**	0.132+/-0.006	0.152+/-0.008	1.16	
**Expt 3 (n = 8–9)**	0.048+/-0.002	0.060+/-0.004	1.24	

Lipid peroxidation assessed by TBARS assay in plasma of transgenic mice treated with 6 weeks of acute hypoxia (AH) showed a significantly increased plasma absorbance levels relative to air control (AC) conditions in 3 repeat assays (mixed modelling analysis on combined data; P < 0.0005). Lipid peroxidation in plasma of positive control WT animals drinking MDA-supplemented acidified water for 6 weeks was significantly increased relative to both AH – (P < 0.01) and AC – (P < 0.001) exposed mice. Mean+/- SEM are shown for each experimental group.

### Effects of acute hypoxia and MDA on primary and metastatic tumour progression

**Figure 3:**
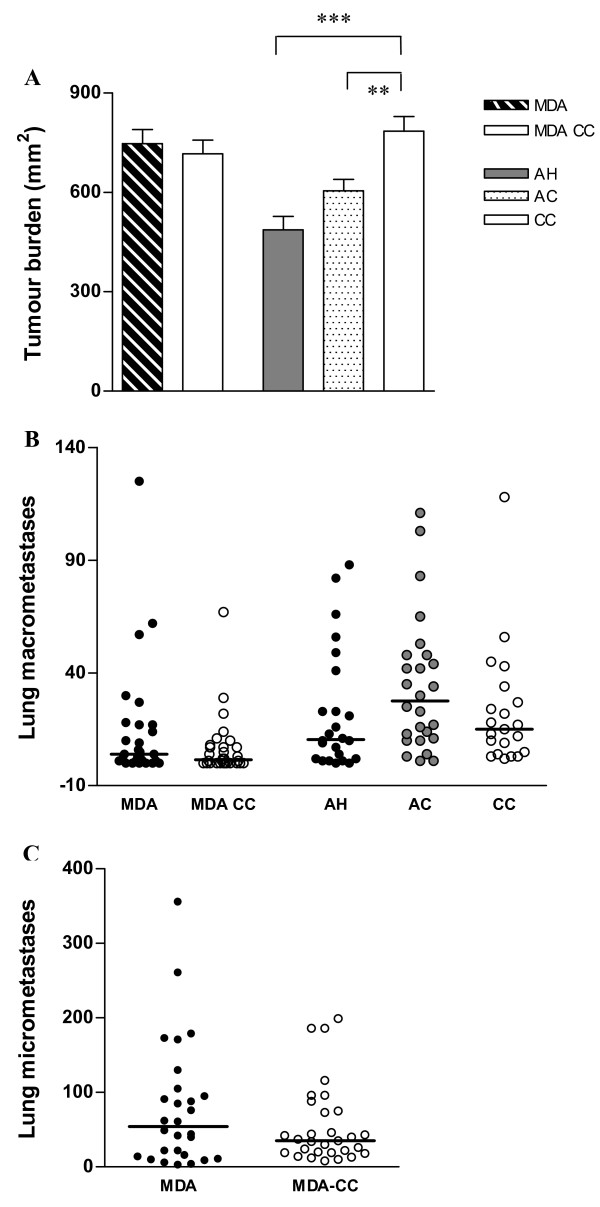
**Primary and metastatic tumour progression in MDA- and AH – exposed mice**. (**A**) CC mice developed significantly larger primary tumours than mice exposed to AH (P < 0.001; ***) or AC conditions (P < 0.01; **). Six-week exposure to MDA did not affect the size of endpoint tumour burden relative to MDA-CC. (**B**) MDA or AH exposure during primary tumour development did not increase macroscopic lung metastases relative to their respective controls. (**C**) A trend towards an increased number of micrometastases was seen following MDA consumption. Micrometastases were not assessed in the hypoxia experiment due to a large number of macroscopic metastases. Abbreviations: MDA = malondialdehyde (n = 28) and MDA-CC = cage controls for MDA group (n = 30), 81 days; AH = acute hypoxia (n = 24), AC = air control (n = 26) and CC = cage controls for AH group (n = 20), 88 days. Bars: mean with SEM (A), medians (B and C). Number of animals used for each group is indicated in parentheses.

To address the question on how MDA or hypoxia would affect tumour progression of the MMTV-PyMT model, primary and metastatic tumour burdens were quantified in mice exposed to these conditions. MDA consumption, which resulted in a significant increase in 8-oxo-dG lesion levels, did not significantly alter any of the tumour progression parameters examined (Figures 3A–C). The primary tumour burden following AH exposure also remained unaffected relative to AC mice, although both AC and AH groups had significantly reduced primary tumour burdens relative to unexposed CC mice (Figure 3A). The numbers of macroscopic metastases showed the same range and no significant differences between the AC, AH and CC groups (Figure 3B).

### Total antioxidant capacity and APE/Ref-1 levels in hypoxia treated mice

**Figure 4:**
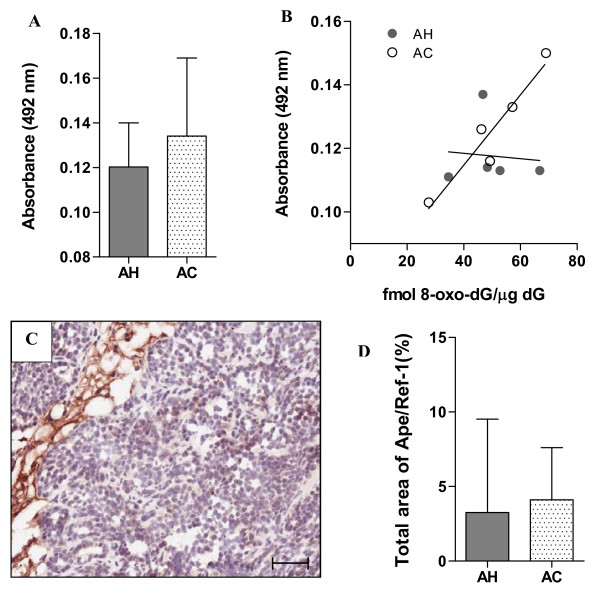
**Total plasma antioxidant capacity and tumour APE/Ref-1 expression**. (**A**) Plasma samples from mice exposed to AH showed a trend of reduced levels of total antioxidant reductive capacity relative to AC mice. (**B**) Scatter plot of total antioxidant reductive capacity and 8-oxo-dG lesions in matching samples showed a significant positive correlation for AC mice (r^2 ^= 0.9094; P < 0.05), which was absent in the AH samples (r^2 ^= 0.008383; P = 0.8836). (**C**) BER protein, APE/Ref-1 was expressed predominantly in the connective tissue of the MMTV-PyMT tumours. (**D**) Total positive staining area for APE/Ref-1 in tumour tissue (excluding connective tissue) did not differ in tissues from acute hypoxia and air control exposed mice. Bars: median with interquartile range. Scale bar: 50 μm.

To determine whether altered antioxidant levels in AH exposed mice may have been the underlying reason for the small increase in 8-oxo-dG lesions, the total antioxidant capacity of plasma from the AC and AH mice was assessed. Plasma from AH-exposed mice showed a trend of reduced antioxidant reductive capacity relative to plasma from AC mice (Figure 4A; P = 0.0667). Assessment of the relationship between plasma antioxidant capacity and tumour 8-oxo-dG lesions revealed a significant correlation between these parameters in the AC group (Figure 4B; r^2 ^= 0.9094, P < 0.05), which did not occur with the hypoxia exposure.

APE/Ref-1 is a multifunctional protein that acts as an endonuclease in the BER pathway and controls the activity of transcription factors, such as AP-1 and HIF-1α by regulating their state of oxidation. Cellular localization of APE/Ref-1 is generally found to be ubiquitous, but varying between nuclear and cytoplasmic compartments [32]. APE/Ref-1 staining in the MMTV-PyMT tumours was primarily localized to the connective tissue (Figure 4C). In tumour cells, the staining levels were generally low and primarily cytoplasmic and were not found to differ between the two treatment groups. Image analysis was conducted in tumour tissues only and in tumour with connective tissue included, but neither analysis showed a difference between AH- and AC-exposed mice (Figure 4D).

### Effects of hypoxia on TAMs and vasculature

**Figure 5:**
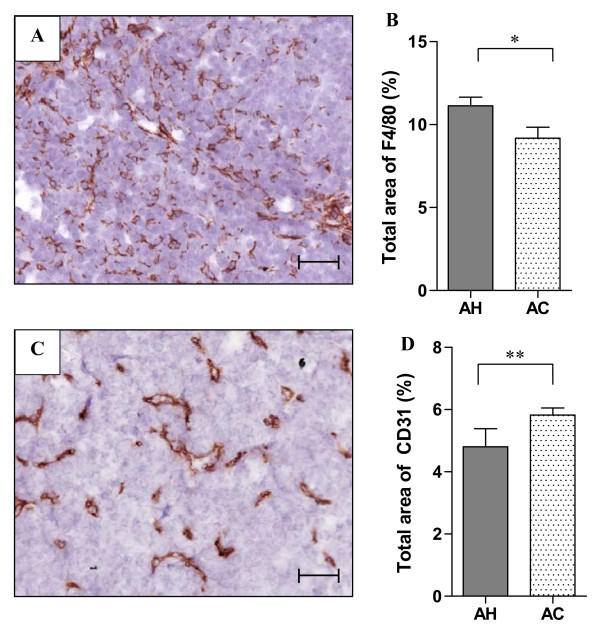
**Quantification of TAM and vascular markers**. (**A**) Representative cytoplasmic expression of MØ marker F4/80 in a MMTV-PyMT tumour section. (**B**) Total positive staining area of F4/80 was significantly higher in AH than AC treated animals (P < 0.05; *). (**C**) Representative staining pattern of vascular marker CD31 in MMTV-PyMT tumours. (**D**) Total CD31 positive area was significantly reduced following hypoxia exposure (P < 0.01; **). AH = acute hypoxia, AC = air control. Bars: mean with SEM. Scale bars: 50 μm.

TAMs have been previously reported to be involved in tumour progression in the MMTV-PyMT model and to accumulate in hypoxic areas [21,25,26]. To quantify TAM content, tumour samples were stained with the MØ marker F4/80. Interestingly, the F4/80 positive staining was seen evenly throughout the tumours in contrast to the highly localized staining pattern commonly seen with extrinsic hypoxia markers such as EF5 (cf. Figures 1A and 5A). The overall levels of F4/80 were significantly increased in samples from hypoxia-relative to air-exposed mice (Figure 5B; P < 0.05). TAMs have been reported to have pro-angiogenic effects [33,34] and therefore tumour vasculature was quantified through CD31 staining (Figure 5C). Similarly to our previous *in vivo *acute hypoxia study in a cervical tumour model the total area of CD31 positive staining was significantly reduced following AH exposure despite the increase in TAMs [35] (Figure 5D; P < 0.01).

## Discussion

Hypoxia has been reported to enhance metastatic tumour progression and to be associated with oxidative stress, genomic instability and TAMs [21,36-38]. We have previously shown metastasis-promoting effects of *in vivo *acute hypoxia in a murine fibrosarcoma and a human orthotopic cervical model [4,35] and in the current study, we examined the relationships between the above parameters in an *in vivo *transgenic breast cancer model with high metastatic potential.

In human breast cancer, the mutagenic end product of lipid peroxidation, MDA, is commonly used as a marker for oxidative stress [19,39], which in turn has been associated with induction of epithelial cell invasiveness and metastatic phenotype [40,41]. In our study, TBARS assay of plasma for lipid peroxidation products from mice exposed to AH shows a significant increase relative to AC mice. The increased level of oxidative stress following AH exposure was further supported by the trend of reduced total antioxidant capacity in the plasma of AH-exposed mice. The effects of increased oxidative stress in the hypoxia-exposed (and in the MDA-fed) animals were also quantified through HPLC analysis of 8-oxo-dG lesions. These lesions are known to induce GC→TA transversions [18] and have been previously used as a measure of oxidative stress induced by hypoxia and as a marker of oxidative stress in breast cancer patients [38,42]. Our HPLC analyses of breast tumour tissue were consistent with the results of the TBARS and antioxidant assays and showed greater numbers of 8-oxo-dG lesions following AH exposure and MDA consumption. We chose to quantify 8-oxo-dG lesions based on their ROS-dependent formation and their ability to induce mutations [18,43]. Therefore, one of our underlying assumptions was that increased 8-oxo-dG lesion levels would provide a general indication of potential DNA lesions and hence mutagenic events associated with oxic stress. An alternate approach might have been to quantify specific, tumourigenesis-modifying mutations such as base pair mutations, gene amplifications, chromosomal translocations or aneuploidy directly from tumour biopsies, which would allow comparison of mutation levels with tumourigenesis data on an individual animal basis. However, identifying which are the critical lesions is problematic and the relevant lesions might be different in different tumours in the same animal, complicating the analysis.

Together, our results provide *in vivo *evidence that the cyclic hypoxia-breathing regime causes significant oxidative stress and might be expected to induce genomic instability and tumour progression. However, despite the increase in oxidative stress we observed little change in tumour progression, although both gassing exposures did significantly reduce primary tumour burdens relative to cage control mice. This is likely due to various extra stressors in the gassed mice such as handling, temporary (4 hr) absence of food and water and exposure to foreign cage mates, conditions that have all been reported to have the potential to modify tumour progression [44-46]. Some of the suggested underlying mechanisms include stressor-mediated changes in various immune cell and cytokine levels, accelerated telomere shortening and apoptosis induction [46-48].

Although the increases in hypoxia-induced lipid peroxidation and 8-oxo-dG lesions were statistically significant, our data suggests that these changes may not have been large enough to induce a biological impact. Therefore, we treated animals with MDA, which induced greater increases in 8-oxo-dG lesions than those seen with the AH exposure, and assessed the animals for primary and metastatic tumour progression. Despite the significant increase in 8-oxo-dG lesions in MDA-fed mice, again no respective changes in tumour progression were observed. These findings suggest that increases in oxidative stress and 8-oxo-dG lesions do not play a significant role in the tumour progression of the MMTV-PyMT model. Similar conclusions have also been reported from experiments with the MMTV-PyMT model where the lack of a significant difference in point mutations between normal, malignant and metastatic tissues and the lack of loss of heterozygosity (LOH) above background levels was attributed to the strong transforming ability of the middle T oncogene [49,50]. This is consistent with the findings that several pathological conditions with increased 8-oxo-dG levels in tissue can occur without tumour incidence and studies that have reported no relationship between DNA adduct levels or even megabase deletions and cancer incidence [51,52]. Furthermore, the mouse model deficient in *OGG1*, a glycosylase responsible for removal of 8-oxo-dG lesions, fails to demonstrate a significant effect on tumourigenesis despite significantly elevated 8-oxo-dG lesions [53,54]. A similar observation has been reported for another glycosylase, *MYH*, and only when the two mutants are combined or another stressor, such as UV exposure, is introduced is an increase in tumour incidence observed [55,56]. Therefore, it is possible that similar compensatory mechanisms could have prevented the hypoxia exposures from translating into changes in tumour progression and that different results could be seen if the gassings were imposed on one of the above-mentioned repair-deficient models. Overall, our findings are consistent with the idea that any effect of oxidative stress on tumour progression in the MMTV-PyMT model would require stress levels, which are not inducible by the degrees of cyclic hypoxia or MDA exposures used in this study, and emphasize the importance of the lesion type, location and context in addition to their number.

The 6-week acute hypoxia exposure also resulted in a significant increase in TAMs as detected by the F4/80 MØ marker. TAMs are thought to represent a distinct MØ population, for which phenotypic characterization remains incomplete. They differ in their activity from the MØ populations found in normal tissues and their differentiation depends on the cytokines expressed in the host tumour [57]. Previous work has implicated a multitude of roles for TAMs in tumour progression, including angiogenesis and stimulation of cellular metastasis and survival [23,58]. Our data show *in vivo *evidence of hypoxia-mediated TAM accumulation to tumours, independent of angiogenesis. Although discordances between hypoxia and stimulation of angiogenesis have been reported previously, the absence of increased angiogenesis with increasing TAM content seems to be in contrast to previous reports [26,59]. This dissimilarity may be due to the different markers used for angiogenic quantification or varying angiogenic potentials between hypoxia- and CSF-1-stimulated TAMs. Although the hypoxia-mediated increase in F4/80 positive MØs did not translate into increased tumour burden or lung metastases in the MMTV-PyMT model, the lack of specific TAM co-localization to hypoxic areas is consistent with previous observations in the MMTV-PyMT model where no co-localization of TAMs to hypoxic areas, as identified through pimonidazole staining, was observed [34]. In future work, the AH gassing exposure could be shifted from the period of primary tumour growth to metastasis formation stage since the role of MØ in tumour progression has been shown to occur at the invasion stage of tumour cells where MØ physically interact with cells invading into the vasculature [26].

Although an *in vivo *study examining the effects of whole body exposure to hypoxia on leukaemia progression reported no significant differences in blood cell counts or cellular proliferation or apoptosis rates of the control mice following daily 18 hour exposure to ~10% oxygen during 30 days, significantly increased levels of the anti-angiogenic factor, thrombospondin-1, have been observed in hypoxia-exposed endothelial cells [60,61]. Therefore, one potential limitation of the current model is that the whole animal in addition to the tumour was exposed to cycling hypoxia, which may induce adaptive physiological responses that might be able to neutralize or mask changes occurring at the tumour level.

## Conclusion

We have demonstrated increased 8-oxo-dG DNA lesions following exposure to acute cyclic hypoxia (AH), supporting the concept that tumour hypoxia could have mutagenic potential. However, neither this exposure or treatment with MDA, which caused the greater increase in 8-oxo-dG DNA lesions, affected tumour progression, raising doubts concerning the importance of oxidative stress in tumour progression of the MMTV-PyMT model. In this context, it is of interest that we have obtained similar results in the MMTV-Neu mammary tumour model (Kalliomäki, 2008). Interestingly we also observed that AH exposure increased TAM content of the PyMT tumours. This increase was not associated with hypoxic areas in the tumours nor did it relate to angiogenesis, one of the putative roles of TAMs. Our current work suggests that this response may be dependent on changes associated with the MMTV-PyMT oncogene since similar hypoxia exposure did not significantly increase the F4/80^+ ^MØ content of the MMTV-Neu tumours (Kalliomäki, 2008).

## Abbreviations

2-NP: 2-nitropropane, 8-oxo-dG: 8-oxo-7,8-dihydro-2'-deoxyguanosine; APE/Ref-1: Apurinic/apyrimidinic endonuclease 1/redox effector factor-1; BER: Base excision repair; BHT: Butylated hydroxytoluene; CAIX: Carbonic anhydrase IX; CXCR4: Chemokine receptor 4; EF5: 2-(2-nitro-1-*H*-imidazol-1-yl)-*N*-(2,2,3,3,3-pentafluoropropyl) acetamide; GLUT-1: Glucose transporter 1; HIF-1α: Hypoxia-inducible factor-1α; HPLC-EC: High performance liquid chromatography with electrochemical detection; IHC: Immunohistochemistry; Ku70: ATP-dependent DNA helicase 2 subunit 1; MDA: Malondialdehyde; MIF: Macrophage inhibitory factor; Mlh1: mutL homolog 1; MMTV: Mouse mammary tumour virus; MØ: Macrophage; MSH2/6: MutS homolog 2/6; O/N: Overnight; PMS2: PMS2 postmeiotic segregation increased 2; PyMT: Polyoma middle T; RAD51: DNA repair protein RAD51 homolog 1; ROS/RNS: Reactive oxygen/nitrogen species; R/T: Room temperature; TAM: Tumour-associated macrophage; TBARS: Thiobarbituric acid reactive substance; VEGF: Vascular endothelial growth factor; Xrcc4: X-ray repair cross complementing protein 4.

## Competing interests

The authors declare that they have no competing interests.

## Authors' contributions

TMK contributed to the design of the experiments, conducted the animal studies and the majority of the statistical analyses, assisted in the HPLC sample preparation and drafted the manuscript. GMcC performed the HPLC analyses and drafted the associated sections of the manuscript. SJL performed part of the hypoxia exposures and critically revised the manuscript. PGW supervised the HPLC studies and critically revised the manuscript. RPH conceived the study, supervised the experiments and helped to draft the manuscript. All authors have read and approved the final manuscript.

## Pre-publication history

The pre-publication history for this paper can be accessed here:


